# Antenatal Care Attendance and Multiple Micronutrient Supplementation Intake: Perspectives from Women and Antenatal Care Service Providers in Rwanda

**DOI:** 10.3390/nu18030373

**Published:** 2026-01-23

**Authors:** Giulia Pastori, Kesso Gabrielle van Zutphen-Küffer, Shashank Sarvan, Yana Manyuk, Elvis Gakuba, Yashodhara Rana, Jack Clift, Kara Weiss, Bonnie Weiss, Xiao-Yu Wang, Aline Uwimana, Claude M. Muvunyi, Eliphaz Tuyisenge, Samson Desie, Melinda K. Munos, Sufia Askari

**Affiliations:** 1 Sight and Life, 4303 Kaiseraugst, Switzerland; kesso.vanzutphen@sightandlife.org (K.G.v.Z.-K.); shashank.sarvan@sightandlife.org (S.S.); yana.manyuk@sightandlife.org (Y.M.); elvis.gakuba@sightandlife.org (E.G.); sufia.askari@sightandlife.org (S.A.); 2 Eleanor Crook Foundation, San Marcos, TX 78666, USA; yrana@eleanorcrookfoundation.org (Y.R.);; 3 CRI Foundation, Boston, MA 02116, USA; 4 Rwanda Biomedical Centre, Kimihurura, Kigali P.O. Box 7162, Rwanda; aline.uwimana@rbc.gov.rw (A.U.); claude.muvunyi@rbc.gov.rw (C.M.M.); eliphazeng@gmail.com (E.T.); 5 UNICEF Rwanda, Kacyiru, Kigali P.O. Box 381, Rwanda; sdesie@unicef.org; 6 Department of International Health, Johns Hopkins Bloomberg School of Public Health, Johns Hopkins University, Baltimore, MD 21218, USA; mmunos@jhu.edu

**Keywords:** micronutrient supplementation, anemia, adherence, prenatal care, implementation research

## Abstract

Background/Objectives: Emerging evidence suggests that multiple micronutrient supplements (MMS) provide additional benefits for maternal and neonatal health compared with iron and folic acid (IFA) supplements. To achieve effective coverage, acceptability, and adherence—and to inform a nationwide rollout of MMS—it is essential to understand the context-specific factors that shape implementation. This study evaluated the pilot implementation of MMS in Rwanda to identify key enablers, areas for improvement, and challenges related to antenatal care (ANC) attendance and MMS use. Methods: Data were collected through a survey of 3257 women who attended ANC services, seven focus group discussions with 35 ANC attendees, and key informant interviews with 20 ANC nurses and 21 community health workers. Results: Pregnant women reported high ANC attendance (74%) and MMS consumption (79%), largely driven by strong motivation and awareness of MMS benefits. Strategies to remember daily intake and to manage side effects supported adherence, as did reminders, motivation, and information from family members and healthcare providers. Limited patient-centered counselling, financial constraints, barriers to accessing ANC services, and product stock-outs were key areas for strengthening service delivery in Rwanda. Conclusions: Sustaining high ANC attendance and MMS adherence as the program transitions from the pilot phase to national scale-up is essential. Improving counseling quality and strengthening supply chains may reinforce ANC services and support sustained MMS adherence, with benefits for maternal and child health.

## 1. Introduction

In Rwanda, maternal undernutrition remains a major public health problem and is associated with maternal mortality and morbidity as well as poor child health outcomes. Although limited data are available on micronutrient deficiencies among women [1], Rwanda presents a high level of malnutrition, with 33% of the children being stunted, 13% of women of reproductive age, and 25% of pregnant women being anemic [2].

Micronutrient supplements containing iron are effective in improving maternal and child health, particularly in reducing maternal iron deficiency anemia [3]. In Rwanda, the provision of iron and folic acid (IFA) supplements is included in ANC policies and guidelines and is delivered as part of standard ANC services at health centers. However, in 2019–2020, only 47% of women attended at least four ANC visits and only 16% reported consuming IFA for at least 90 days during pregnancy [2]. This may reflect low ANC attendance, limited IFA availability at ANC visits, low adherence to IFA, or a combination of these factors—highlighting challenges at the health-system level.

Multiple micronutrient supplements (MMS) offer additional benefits compared to IFA in terms of birth outcomes, including higher birth weight, a reduced risk of being small for gestational age, decreased stillbirth and preterm birth rates, and early signs of stronger brain development [4]. These benefits appear greatest among anemic and underweight pregnant women, and among women who initiate supplementation earlier and adhere throughout pregnancy [5,6]. The standardized formulation of MMS is the United Nations International Multiple Micronutrient Antenatal Preparation (UNIMMAP), which contains iron, folic acid, and 13 other essential vitamins and minerals [7]. This formulation helps address increased micronutrient requirements during pregnancy.

Given the global evidence base [8,9] and Rwanda’s national strategy to reduce stunting by improving maternal and child health (2024–2029), the Government of Rwanda has decided to introduce MMS in public ANC services. To support the transition from IFA to MMS, a pilot program was implemented in seven districts in 2024. Implementing and evaluating a pilot before nationwide adoption provides an opportunity to understand the challenges faced in the delivery and consumption and to generate insights essential for effective and sustainable scale-up.

During the pilot, MMS was distributed to pregnant women during their ANC visits at the health center, as is the case for IFA, by nurses and midwives in charge of ANC services (referred to hereafter as ANC nurses). The ANC guidelines were updated, replacing IFA with MMS, and ANC nurses were trained to provide standard counseling on MMS. The program was also envisioned as an entry point for strengthening the broader ANC system.

While MMS has demonstrated benefits for maternal and newborn outcomes, context-specific factors affecting implementation must be understood to ensure effective coverage, acceptability, and adherence—particularly as Rwanda transitions from IFA to MMS. To support Rwanda’s transition and strengthen ANC service delivery, this study evaluated the pilot to identify key enablers, areas for improvement, and challenges experienced by pregnant women and ANC providers related to ANC attendance and MMS use.

## 2. Materials and Methods

This cross-sectional mixed-methods study combined a quantitative survey and qualitative interviews to evaluate the MMS pilot among pregnant women attending ANC services, ANC nurses, and community health workers (CHWs) in seven Rwandan districts where MMS distribution started in January 2024. The selected districts—Gasabo (City of Kigali); Gicumbi, Musanze, Burera (Northern Province); and Nyabihu, Rutsiro, Ngororero (Western Province)—were chosen due to their high stunting rates. In each district, five health centers were randomly selected (35 centers total).

The qualitative component aimed to gather in-depth perspectives from beneficiaries and providers to inform recommendations for MMS scale-up. Data collection tools were developed in English and translated into Kinyarwanda. Trained data collectors conducted data collection between December 2024 and January 2025.

### 2.1. Study Population and Sampling

The survey targeted 3500 adult women who were randomly selected from the ANC registers of pregnant women who had their first ANC contact between April and September 2024 at selected health centers. From each health center, 100 adult women were selected. Adult women were targeted as they constitute the majority of pregnant women in the study areas. Women living outside the study areas, younger than 18 years old, and/or with complications or pathologies were excluded at the time of selection from the ANC register.

Among the surveyed women, four to six women were selected for each Focus Group Discussion (FGD). On the service-delivery side, 20 ANC nurses and 21 CHWs were selected from three of the five randomly selected health centers and their corresponding catchment areas. These participants were purposively selected for their key roles in maternal and infant care at the health center and community levels, respectively.

### 2.2. Data Collection Tools

To collect information on socio-demographic characteristics, coverage, adherence, barriers, enablers, and acceptability of MMS, a structured survey was administered. The Demographic and Health Survey (DHS) questionnaire was used to develop survey questions. The survey included dichotomous and multiple-choice questions and was administered by trained data collectors using KoboToolbox software (version 2025.3.3) [10].

To explore underlying reasons for identified barriers and enablers, as well as specific needs, and possible solutions, seven focus group discussions (FGDs) were conducted: four FGDs included women who consistently consumed MMS (Doers), and three involved women who did not (Non-doers) to identify specific drivers of adherence. Doers and Non-doers were purposively selected based on MMS adherence and then randomly selected from each group.

To capture service providers’ perspectives on challenges, needs, and best practices, 41 key informant interviews (KIIs) were conducted, except in Rutsiro, where ANC nurses were unavailable. Semi-structured guides with open-ended questions and probes were used for both FGDs and KIIs.

All data collection tools were piloted and refined before the main data collection.

### 2.3. Data Processing and Analysis

Survey data were checked for completeness and inconsistencies. Any incomplete, duplicated, and inconsistent entries were identified and, if necessary, removed. Descriptive analysis was conducted to characterize the surveyed women and assess coverage, adherence, and acceptability of ANC and MMS (Appendix A Table A1). For continuous variables, means and standard deviations (SD) were calculated for normally distributed data, while medians and interquartile ranges (IQR) were used for non-normally distributed data. Categorical variables were summarized using percentages and frequency tables. Analyses were performed using R software (version 4.3.1) [11].

### 2.4. Qualitative Data Analysis

Based on the reported number of days consuming MMS over the last seven days, the women were classified into Doers (7 days) or Non-doers (<7 days). All interviews—FGDs and KIIs—were anonymized, transcribed verbatim in Kinyarwanda, and translated into English. Two researchers independently analyzed transcripts using pre-specified coding themes based on the interview guides and research objectives, informed by a grounded theory approach [12]. Areas of disagreement were discussed and reconciled through collaborative refinement of the coding. Topical codes were used to categorize quotations according to interview-guide domains, and open interpretive coding was used to identify emerging themes within and across topical areas. Quotations were selected to illustrate key themes, with respondents’ roles indicated. FGDs were analyzed separately for Doers and Non-doers to identify similarities and differences between users and non-users. The analysis was performed using NVivo (version 15) [13].

### 2.5. Ethics

The study was approved by the National Ethics Committee of the Republic of Rwanda (RNEC 638/2024). Informed consent was obtained in Kinyarwanda from each respondent before the interviews.

## 3. Results

The 3257 women surveyed (93% response rate) were, on average, 29 years old, and lived in a household with four people and two children (Table 1). The majority were married (87%), employed in farming activities (64%), and their highest level of education was primary school (59%).

### 3.1. ANC Attendance and MMS Counseling

**Figure 1:**
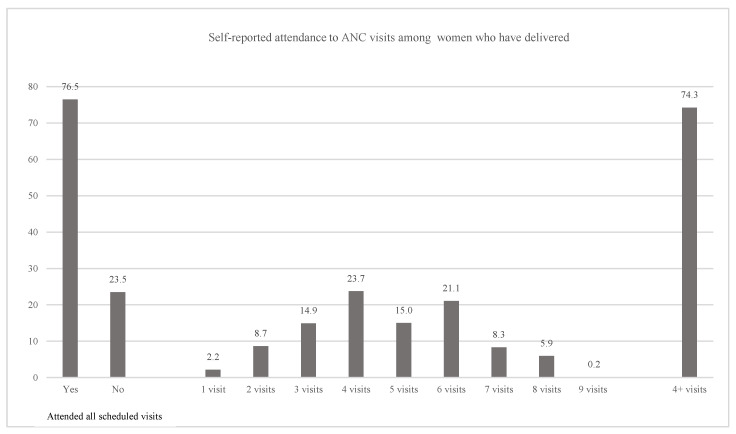
Antenatal care (ANC) attendance among women who had already delivered at the time of the survey (*n* = 1184): Proportion who reported attending all scheduled ANC visits (left); distribution of the self-reported number of ANC contacts attended (middle); and proportion who attended at least four ANC contacts (right).

ANC attendance was assessed among women who had delivered by the time of the survey to capture attendance across the full pregnancy. Most women reported having attended all the ANC visits that were scheduled (77%) and 74% of the women attended at least four ANC visits (Figure 1).

**Figure 2:**
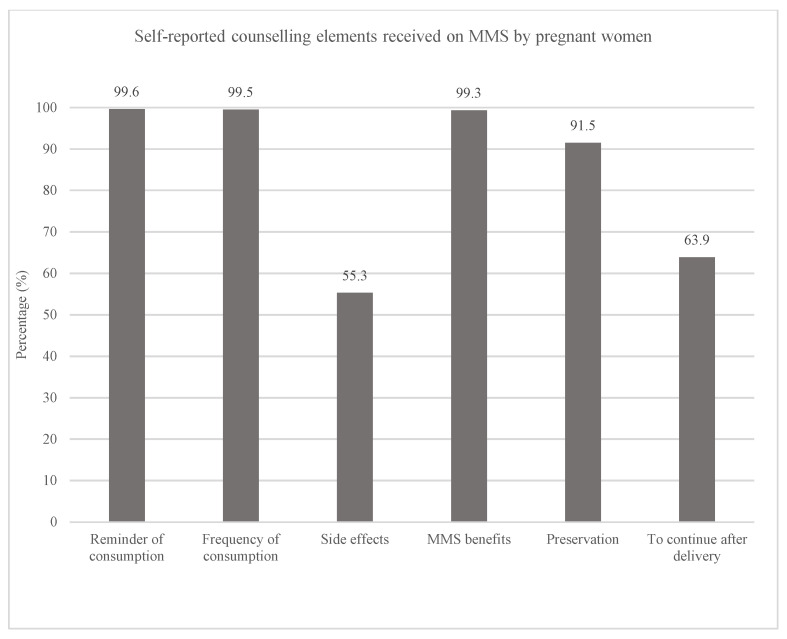
Proportion of pregnant women who received each element of the counselling on MMS (*n* = 2073).

During ANC visits, 44% of the pregnant women reported receiving complete counseling on MMS. Specifically, all pregnant women said that they were reminded to consume MMS and were counselled on the frequency of consumption; the majority of them were informed about its benefits (99%) and advised on how to store the MMS (91%) (Figure 2). In contrast, 55% of the pregnant women received information on side effects and 64% were advised to continue consuming MMS after the delivery.

Women mainly reported attending ANC services due to habituation from previous pregnancies (33%), and strong social support from the partner (31%), other family members (11%), and CHWs (10%). Conversely, the main reasons for not attending all scheduled ANC contacts were lack of awareness of being pregnant (22%), no follow-up contact scheduled (20%), lack of time (18%), forgetfulness (12%), transportation issues (5%) and long distance to the health facility (4%).

### 3.2. Coverage and Adherence to MMS

**Figure 3:**
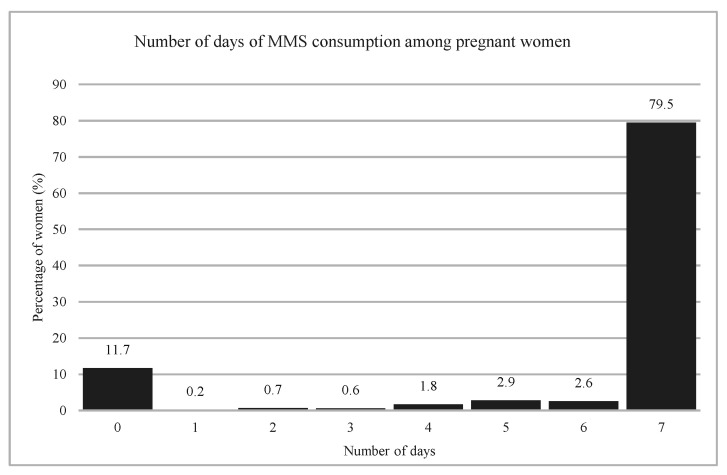
Distribution of the number of days on which MMS was consumed in the last 7 days by pregnant women at the moment of the survey (*n* = 2073).

**Figure A1:**
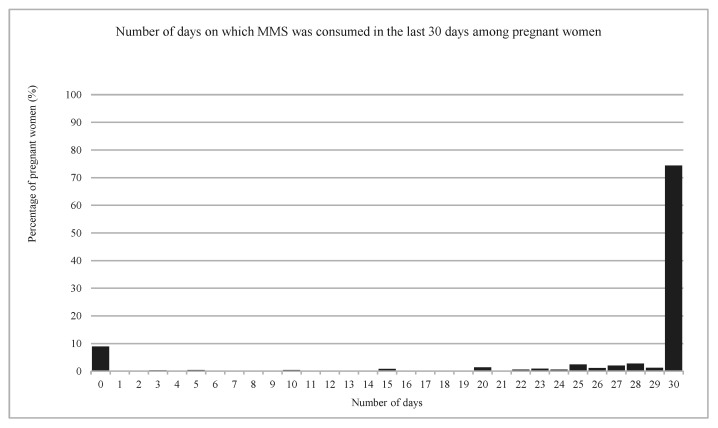
Distribution of the number of days on which MMS was consumed in the last 30 days, by pregnant women at the moment of the survey (*n* = 2073).

**Figure A2:**
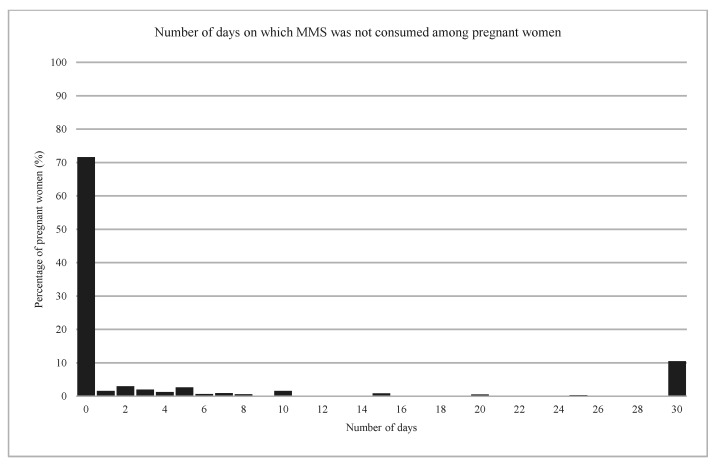
Distribution of the number of days on which MMS was not consumed in the last 30 days, by pregnant women at the moment of the survey (*n* = 2073).

All surveyed women reported having received MMS during their current or last pregnancy, and the median (IQR) total number of tablets received was 180 (119–180). Among women who were pregnant at the time of the survey, most reported consuming MMS every day (80%) or not at all (12%) in the previous seven days (Figure 3), with a median (IQR) of seven days (0–7). The distribution of supplement consumption in the previous seven days was consistent with the distribution of the 30-day recall (Appendix A Figure A1) and of the non-consumption days (Appendix A Figure A2).

**Figure 4:**
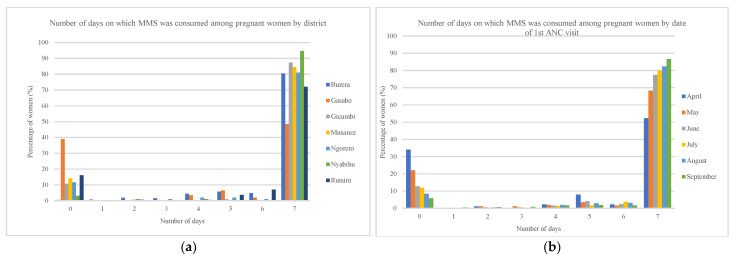
Distribution of the number of days on which MMS was consumed in the last 7 days by pregnant women at the moment of the survey (*n* = 2073): (**a**) by district; and (**b**) by date of their first ANC visit.

The pattern of consumption of MMS was similar across districts (Figure 4a), except in Gasabo district, where a higher proportion of women reported zero days of consumption in the last seven days compared to other districts because of stockouts. MMS consumption declined as pregnancy progressed (Figure 4b), using date of first ANC contact as a proxy for gestational age.

The majority of the women reported liking the supplements (95%) and expressed motivation (41% *very motivated*; 56% *motivated*), confidence (49% *very confident*; 49% *confident*), and satisfaction (43% *very satisfied*; 55% *satisfied*) with supplement consumption. They mainly reported feeling motivated because of the information received (98%) and were *very satisfied* (50%) and *satisfied* (48%) regarding the information received about MMS. Among the surveyed women, 98% reported receiving some form of family support for MMS consumption.

The majority of the women found it *very easy* (51%) or *easy* (46%) to incorporate MMS consumption in their daily routine mainly because they were habituated (75%), took it with a meal (10%), were reminded by someone (6%), always took it at the same time (4%) or set an alarm (4%). By contrast, 22% reported stopping MMS, mainly because they ran out of tablets (22%), forgot (20%), experienced side effects (16%), or stopped after giving birth (15%).

### 3.3. Enablers of ANC Attendance

#### 3.3.1. Trust and Reassurance

Most FGD participants were motivated to attend ANC services for reassurance about their pregnancy and to receive guidance on their own and their baby’s health. The comfort of regular monitoring and timely advice and action to ensure a healthy pregnancy was a common theme (Table 2).

*“When I find out that I am pregnant, I rush to the health centre, and I really believe that the nurse will test me and give me the answer and start to monitor me and the baby.”*.

(Woman, Doer)

In addition, trust in the reliability of CHWs’ and health professionals’ guidance was a key motivator for attending ANC services.

*“Our community health worker is my neighbor, so she comes home more often and every time she comes, she asks me when I will go back, when is my appointment and she reminds me when my appointment is due.”*.

(Woman, Non-Doer)

#### 3.3.2. Reminders and Follow-Ups

ANC nurses reported making follow-up calls for missed appointments, while CHWs identified home visits as effective for improving ANC attendance and MMS adherence. Women valued these contacts as essential for continuity of care.

*“We visit them and teach them in community meetings telling them that when a woman becomes pregnant, she should visit a health center for all recommended ANC visit and checkups, feeding on balanced diet following foods and crops available in our areas of living, teach them about saving schemes in order to get prepared for welcoming the newborn while having minimum facilities.”*.

(CHW)

#### 3.3.3. Support from Family and Community

Partners’ absence and lack of support during ANC visits, often due to work duties, perceived negligence or family conflict were reasons for delaying or skipping appointments.

*“There are conflicts in the house: when you have a problem with your husband, there are times when you cannot participate in the programs that are planned.”*.

(Woman, Doer)

*“I also notice that some pregnant women do not attend services provided for expectant mothers because they fear or their husbands have jobs far away. They don’t go for check-ups, and it is a requirement that a pregnant woman must come for her first check-up with her husband. This makes the woman wait for her husband until he returns home, and then they go together to the health facility.”*.

(Woman, Non-Doer)

CHWs emphasized the significance of community involvement, such as peer support. Community meetings were key platforms for sharing information and providing support. However, CHWs mentioned that older women tend to go less to the ANC service because of previous experiences.

*“Other than CHWs, even other pregnant women encourage their neighbors to attend ANC, after coming here to the health center and learn about the importances of attending ANC visits, they encourage others.”*.

(CHW)

### 3.4. Barriers to ANC Attendance

#### 3.4.1. Financial Constraints

Both Doers and Non-Doers reported financial barriers, such as lack of health insurance and transportation costs, as challenges to attending ANC contacts. ANC nurses and CHWs echoed the same challenges: particularly, the costs for transportation and service-related costs. Women reported being unable to pay for services or needing to prioritize work over attending appointments. Moreover, some women were discouraged by the number of ANC contacts they were expected to attend.

*“One of the reason some parents delay seeking medical services is that obtaining health insurance (mutuelle) is challenging, especially for those trying to change their social category.”*.

(Doer)

#### 3.4.2. Physical Barriers

Women faced physical barriers, such as transportation difficulties during the rainy season, and time constraints. On average, women estimated that it took one hour to reach the health centre.

*“There are also women who face challenges due to coming from far, which prevents them from attending on time, or there may be issues like rain and others. Finding enough time is challenging due to the heavy workload because there are also few staff members.”*.

(ANC nurse)

*“During heavy rainfall, pregnant women or Women who have given birth both face difficulties in finding transportation to the health center.”*.

(Woman, Doer)

#### 3.4.3. Emotional Barriers

Women reported emotional barriers such as feeling ashamed and judged by health care providers for having several subsequent pregnancies, lacking trust in modern medicine and reporting negative service experiences. These often led to delaying or skipping ANC services.

*“Sometimes, adolescents that became pregnant don’t usually attend ANC or any other service because they are afraid to go out and they think people are going to judge them. Also, some women in this community have different beliefs.”*.

(Woman, Non-doer)

### 3.5. Factors Influencing MMS Consumption

#### 3.5.1. Motivation for MMS Consumption

Doers were aware of and motivated by the benefits of consuming MMS. The most reported benefits were that MMS increased “the level of blood” in women’s bodies, the baby would be born healthy and with a healthy weight, and they would deliver without complications. Women also reported experiencing an increase in energy and appetite as a benefit of consuming MMS.

*“What motivates us to use MMS tablets is that they level up my blood, the baby grows well in the womb, and the child’s body and organs develop properly.”*.

(Woman, Doer)

Most Doers reported strong belief in the supplement’s benefits for themselves and their babies, which supported continued use despite initial challenges. In contrast, Non-doers often reported low perceived need for supplementation and limited belief in short-term benefits, suggesting lower motivation. Some also displayed optimism bias, citing positive birth stories without MMS use, showing a lack of intrinsic perceived need. ANC nurses similarly noted that the absence of immediate benefits discouraged adherence:

*“The importance I have seen: if you follow it properly, you will give birth without surgery, the doctor used to tell us the importance of it and how to use it, a person who takes the pills correctly will give birth to a child without complications, it will make the child give birth without any problems, which will make the child grow well in the womb, without birth defects.”*.

(Woman, Doer)

*“I don’t take them regularly because I forget, and sometimes I feel like I can go without them.”*.

(Non-doer)

*“They told us things that might happen when we don’t take them, like is it mandatory to face all those problems that we were told? Like this is my fourth child and I didn’t take any tablets even those ones that we had before and yet my children don’t have any problem.”*.

(Woman, Non-doer)

The perceived benefits of MMS reported by ANC nurses and CHWs closely aligned with those expressed by the women, such as “increased blood”, energy and the baby’s overall health. In addition, they highlighted the health benefits and impact of consuming MMS on the community as a key motivator for them to encourage women to consume the supplement.

*“The benefit is that a mother who uses MMS improves her health and the health of her child. I have noticed a difference.”*.

(ANC nurse)

According to CHWs, MMS was well accepted by the women due to the tangibly experienced benefits on birth outcomes and mothers’ health. A few CHWs reported the misconceptions and negative opinions among some women, such as preferences towards traditional medicine, lack of perceived value, and knowledge surrounding the benefits.

*“Some of the women in this community who went of the church developed wrong beliefs that going to the hospital is bad for their health because they use artificial materials, and they don’t believe in modern medicine they think traditional medicine is better.”*.

(CHW)

#### 3.5.2. Side Effects

Non-doers cited side effects as a primary reason for discontinuing MMS. Doers, however, did not consider side effects as a deterrent or found coping strategies to deal with them, which helped them continue consumption. Some Doers also attributed their lack of side effects to following the health professionals’ instructions correctly.

*“It once caused nausea, but I later realized it was because I took it without eating”*.

(Woman, Doer)

*“The only side effect I had from those tablets was that I would take them and then vomit. However, once I stopped taking them, I didn’t experience any other issues. Whatever was happening to me stopped.”*.

(Woman, Non-doer)

ANC nurses also recognized the side effects as a challenge for some women:

*“The barriers I perceive that might prevent pregnant women from valuing MMS include taking them everyday, some say that they cause nausea and to overcome these barriers I keep explaining them on benefit of taking MMS mainly for having the healthy baby.”*.

(ANC nurse)

#### 3.5.3. Forgetfulness

When asked about difficulties in consuming MMS, both Doers and Non-doers reported forgetting to take the supplement. However, Non-doers described forgetfulness as a direct reason for non-use or discontinuation, whereas Doers described strategies to support daily intake—such as taking MMS at the same time each day (e.g., with meals), keeping tablets by the bedside, or asking a partner or family member to provide reminders.

*“Put the MMS in front of my bed to remind me to eat them. When I go to bed, I always look at them. When I wake up, I always look at them.”*.

(Woman, Doer)

*“I don’t have a specific place to keep them, so I often overlook them when taking my other medicine.”*.

(Woman, Non-Doer)

#### 3.5.4. Family and Community Support

Both Doers and Non-doers emphasized the importance of family support, particularly from partners and healthcare providers, in facilitating MMS consumption. This support often took the form of daily reminders. Some women also noted that partner awareness of the benefits of MMS and the need for adherence was important to their continued use. However, family conflict was concurrently identified as a barrier to consuming MMS.

*“Yes, we are greatly supported by our family members, as they even remind us, which motivates us to take it daily.”*.

(Woman, Doer)

Some Non-doers noted that their partners stopped reminding them after they discontinued taking the supplement. In some cases, partners were absent during ANC contacts due to work duties or negligence.

*“My husband supported me, he would always remind me and tell me that since the doctor prescribed it, it must be important, and I should be patient and take it. But eventually, it became overwhelming”*.

(Woman, Non-doer)

ANC nurses also recognized support from partners and CHWs as effective strategies for MMS. Many expressed confidence in their ability to remind women to consume MMS during each visit.

*“Collaborating with community health workers to deliver it to our homes would make it easier for us.”*.

(Woman, Doer)

*“The most effective approach we see is regular home visits to remind them, because sometimes, when we arrive, we find that they’ve stopped taking the supplements. They often ask us, “I’ve taken the supplements, what should I do now?” Without our visits, they wouldn’t inform us. I believe frequent visits yield positive results.”*.

(CHW)

Reminders from health professionals were frequently reported to facilitate consumption of the supplements. Women trusted their recommendations and encouragement for taking MMS consistently, and valued reminders given during ANC contacts and home contacts by the CHWs.

*“Doctors always remind us to take MMS tablets every time we visit the hospital.”*.

(Woman, Doer)

*“And since they tell us that a person who drinks it gives birth to a healthy, full-weight, healthy baby, it’s a benefit to us.”*.

(Woman, Doer)

#### 3.5.5. Product Characteristics

Both Doers and Non-doers found product characteristics (taste, smell, size) challenging. For Non-doers, these characteristics were cited as reasons for non-consumption or discontinuation; for Doers, they were obstacles but not deterrents. Almost all surveyed women reported liking the supplements’ packaging (97%). Doers did not suggest changes to packaging, whereas some Non-doers suggested translating package information into Kinyarwanda. Many women strongly suggested changes to the tablets’ characteristics, such as size, color, taste, and smell.

*“Changes that we want include to scentless tablets and change color.”*.

(Woman, Doer)

Overall, perceptions of MMS varied widely among the interviewed women, in terms of taste, smell, color and size. ANC nurses confirmed that some women did not like the smell and the taste of the MMS tablets. The packaging was also criticized for its resemblance to HIV/AIDS medication.

Among women who had consumed IFA during a previous pregnancy, some reported that IFA had an unpleasant smell and caused side effects, while others reported no noticeable smell or taste. Many women perceived MMS as having a stronger smell and aftertaste than IFA. There was consensus that MMS tablets were larger than IFA tablets and that the quantity received was higher, which felt overwhelming for some women.

*“How you would be affected by Iron and Folic Acid is the same as how you would be affected by MMS. The first two were small, they were easy to swallow, the MMS tablets are big and they require the use of something else, such as gum, etc.”*.

(Woman, Doer)

#### 3.5.6. Expectation and Management of Side Effects

Many Non-doers cited insufficient or poor-quality information and limited perceived support from healthcare providers as reasons for confusion and discontinuation or non-resumption of MMS.

*“I completely stopped taking them; I couldn’t handle it, so I threw them away. They had explained that even after giving birth, one should continue taking them, but I didn’t know that. If they had explained it to me properly, maybe I would have tried to continue or waited until after giving birth to resume.”*.

(Woman, Non-doer)

To improve MMS uptake and service provision, many women—both Doers and Non-doers—recommended clearer information on MMS benefits and how MMS differs from other supplements.

Many ANC nurses emphasized the importance of their knowledge of MMS in facilitating its consumption, as their beliefs affect women’s decisions. According to them, a clear understanding of the benefits and proper instructions were key factors in ensuring adherence to MMS consumption. They reported that asking women to repeat instructions during visits helped reinforce retention.

*“Yes, it will have positive impacts because patients always understand everything health care provider tell them. And also, health care providers understand well the importances of MMS and I think it will help in explaining them to the mothers.”*.

(ANC nurse)

#### 3.5.7. Limited Time for Counselling

Most ANC nurses reported providing general information on healthy eating and MMS benefits during routine contacts; however, limited consultation time (15–30 min) often constrained more detailed discussions. This concern was echoed by some women, who felt inadequately informed about MMS consumption.

Limited staff availability was the primary challenge reported. Nearly all ANC nurses cited insufficient personnel to manage patient volume and extended record-keeping, impacting information quality and increasing wait times. Additionally, service disruptions and untrained replacements due to staff turnover were noted as further constraints.

*“It depends on the number of people who come. When there are many, I try to use the time by splitting it among them so that I can attend to everyone. I don’t skip or overlook anything; instead, I provide brief explanations or address them collectively in general to save time”*.

(ANC nurse)

#### 3.5.8. Motivation of ANC Nurses

All interviewed ANC nurses expressed motivation and confidence in counselling on MMS, driven by their belief in its benefits for maternal and infant health. Observing positive outcomes among women under their care reinforced this motivation. Training was the primary source of confidence, while those less confident cited a need for additional training.

*“I am committed. It makes me happy when a woman is healthier and the baby is born healthy, so I encourage them to take it.”*.

(ANC nurse)

Beyond individual health benefits, the supplement’s broader societal impact emerged as a common motivator for ANC nurses to provide MMS. They emphasized that taking MMS supports the wellbeing of families, strengthens communities, and contributes to the country’s future.

*“First of all, the child will be born without any issues related to malnutrition, and the mother will not have any blood-related problems. Therefore, in a broader context, it helps us have a population with good health”*.

(ANC nurse)

## 4. Discussion

This study identified multiple factors influencing ANC attendance and MMS consumption in Rwanda. Most pregnant women reported consistent MMS use, largely driven by strong motivation and awareness of benefits for their own and their babies’ health. Additionally, while opinions on the tablets’ smell, taste, and color varied, most women found the tablets and packaging acceptable. Family and health care providers at the health center and community levels were influential in promoting adherence to MMS and ANC, with reminders, motivation, and knowledge. System-level constraints—including limited patient-centered counseling, financial barriers, and challenges accessing services—highlight entry points for strengthening ANC delivery during the transition from IFA to MMS.

Currently, several countries are transitioning from IFA to MMS, but published evidence on country experiences with transition and rollout remains limited [14]. Rwanda is in the implementation phase with a pilot, as recommended for the transition [15]. Our evaluation highlighted actionable lessons for scale-up, including apparent enablers of high adherence and priority areas for improvement.

Consistent with previous studies, physical and financial barriers limited access to ANC services, and low counseling quality and stock-outs constrained supplement uptake in multiple settings [16,17]. In the context of Rwanda, the lack of available stock at the health center level explained the difference in consumption between districts found in our study. Sustained supply to ensure product access is important for the sustainability of the program [18]. Although this assessment did not identify root causes of stock-outs, attention across the supply chain—including financing, quantification and forecasting, procurement, and distribution—is needed to ensure continuous access.

In contrast to factors previously reported to affect IFA consumption [19,20], ANC nurses and CHWs in our study were strongly motivated and committed to providing MMS and explaining its benefits, which may have supported adherence in our study population. However, limited staffing for ANC services remained a major barrier, consistent with Labonté et al. [16].

Strengthening the support system provided by family members, ANC nurses, and CHWs is essential for promoting and sustaining ANC attendance and MMS adherence. Women highly valued regular reminders and social support, including community endorsement. Similar findings have been reported in Bangladesh [17] and South Africa [21] where family support and community engagement facilitated MMS use. In Rwanda, —where adherence was already high—reinforcing these support systems may yield modest additional gains, particularly by addressing forgetfulness, a common reason for discontinuation. Research on Social and Behavior Change (SBC) strategies could identify feasible, cost-effective approaches to further improve adherence by strengthening pregnant women’s self-efficacy and motivation.

Another key opportunity is building capacity for consistent, patient-centered counseling—particularly on expectations, side-effect management, and adherence strategies—to increase and sustain MMS use. As in our study, counseling quality has been shown to influence adherence among Cambodian women [16]. Although ANC nurses were motivated to counsel women on MMS, short consultations due to staff shortages often limited meaningful engagement. Counseling tended to focus on the product rather than supporting women’s confidence and motivation, and staffing constraints have similarly affected supplement intake in Bangladesh [22] and Haiti [23]. Introducing patient-centered counseling could help women anticipate and manage side effects, set intake goals, and receive follow-up support, alongside efforts to optimize counseling time [7,24].

Counseling should also clearly address postpartum continuation of MMS. Many women discontinued MMS after delivery because they were unaware that continued use postpartum may be possible. This likely reflects gaps in policies and guidelines, which should be updated to cover postnatal care and lactation. In addition, training for ANC nurses should incorporate updated guidance for pregnant and lactating women to support continuation after delivery.

Systemic barriers to ANC attendance—including financial constraints, transportation difficulties, and work obligations—also remain important. Even though MMS was provided free of charge, ANC services were not, and the lack of health insurance reduced access for some women. Expanding health insurance coverage and financial assistance may improve utilization [25,26]. Future research should assess the feasibility and cost–benefit of such approaches, particularly given Rwanda’s shift from four to eight recommended ANC contacts in line with WHO guidance [7]. Additionally, strategies to improve physical accessibility should be explored.

This study’s strengths include its mixed-methods design and inclusion of both service recipients and providers, enabling triangulation of quantitative patterns and qualitative explanations. Key limitations include reliance on self-reported adherence, which may be influenced by recall and social desirability bias. However, the consistency across recall periods suggests recall bias may not have been substantial, although social desirability bias cannot be ruled out. Finally, the focus on seven districts—selected for high stunting prevalence—may limit generalizability to the national context.

## 5. Conclusions

This study highlights the importance of incorporating perspectives from pregnant women, ANC nurses, and CHWs to strengthen ANC attendance and MMS use in Rwanda. By addressing identified challenges and reinforcing existing enablers, ANC attendance and MMS adherence may improve further during the national transition from IFA to MMS.

Sustaining high levels of ANC attendance and MMS adherence as the program moves from the pilot phase to national scale-up will be important to maximize health impact for mothers and babies. Improving counseling quality and strengthening supply—along with ensuring funding and ongoing capacity building—will support long-term program sustainability. Implementing these recommendations may strengthen ANC services and contribute to improved maternal and child health.

## Figures and Tables

**Table 1:** General characteristics of the surveyed women.

	Surveyed Women (*n* = 3257)
Age, mean years (sd)	28.8 (6.8)
Number of children, mean (sd)	1.9 (1.6)
Household size, mean (sd)	3.9 (1.7)
Marital status, *n* (%)	
Married	2842 (87.3)
Partnership	41 (1.3)
Single	305 (9.5)
Separated	45 (1.4)
Divorced	14 (0.4)
Widowed	10 (0.3)
Occupation, *n* (%)	
Farming	2053 (63.0)
Work for wage	404 (12.5)
Work in own business	250 (7.7)
Unpaid family business	157 (4.8)
Unemployed	376 (11.5)
Student	17 (0.5)
Education level, *n* (%)	
No formal education	636 (19.4)
Primary school	1930 (59.3)
Lower secondary	430 (13.2)
Upper secondary	211 (6.5)
Vocation training	28 (0.9)
Tertiary (Degree, diploma)	22 (0.7)

sd: standard deviation.

**Table 2:** Summary of factors of ANC attendance and MMS consumption.

	Factors
ANC Attendance	Trust and reassurance from health professionals Follow-up calls and home visits by Community Health Workers Partner and family support Habituation from previous pregnancies Financial constraints (lack of health insurance, transport costs) Physical barriers (distance, rain, transport issues) Emotional barriers (shame, fear of judgment) No follow-up contact scheduled Lack of time Forgetfulness
MMS Consumption	Strong motivation and awareness of benefits Perceived health benefits ("increased blood", healthy baby, energy) Daily reminders from family and partners Reminders from healthcare providers and Community Health Workers Establishing daily routines Taking MMS with meals Coping strategies for side effects Family support Product stockouts Forgetfulness Side effects Stopping after delivery Product characteristics (size, taste, smell, color) Limited quality of the counseling Insufficient information on side effect management Limited consultation time Lack of perceived immediate benefits Family conflicts
Healthcare Provider Level	Strong motivation of ANC nurses and Community Health Workers Belief in MMS benefits for community health Training on MMS Home visits by Community Health Workers Patient-centered approaches Limited staff availability Staff turnover and untrained replacements Time constraints for detailed counseling Extended record-keeping requirements

## Data Availability

The raw data supporting the conclusions of this article will be made available by the authors on request.

## Abbreviations

The following abbreviations are used in this manuscript:

ANC	Antenatal Care
CHW	Community Health Worker
DHS	Demographic Health Survey
FGD	Focus Group Discussion
IFA	Iron and Folic Acid
IQR	Interquartile Range
KII	Key Informant Interviews
MMS	Multiple Micronutrient Supplement
SBC	Social Behavior Change
UNIMMAP	United Nations International Multiple Micronutrient Antenatal Preparation
WHO	World Health Organization

## Appendix A

**Table A1:** Definition of key indicators.

Indicator	Definition
Coverage	Number of women who reported receiving MMS during their current or latest pregnancy
Adherence	Number of days on which MMS was reported to be consumed in the last 7 and 30 days.
